# Recent Trends in Plastic Surgery: A Network Analysis of the Abstract Titles of the Largest German Plastic Surgery Congress 2023

**DOI:** 10.7759/cureus.60761

**Published:** 2024-05-21

**Authors:** Ron Martin, Jonas Roos, Matthias R Mücke, Frank Siemers, Robert Kaczmarczyk

**Affiliations:** 1 Clinic for Plastic and Hand Surgery, Burn Center, BG Klinik Bergmannstrost, Halle, DEU; 2 Department of Orthopedics and Trauma Surgery, University Hospital of Bonn, Bonn, DEU; 3 Department of Dermatology and Allergy, School of Medicine, Technical University of Munich, Munich, DEU

**Keywords:** dgpraec, conference, plastic surgery, big data, digital health, network analysis

## Abstract

Background: Every year, German-speaking experts in plastic, reconstructive, and aesthetic surgery gather to discuss the latest developments at Germany's largest conference for plastic surgery, the joint annual meeting of the German Society of Plastic, Reconstructive and Aesthetic Surgery (DGPRÄC) and the Association of German Aesthetic Plastic Surgeons (VDÄPC). Since the topics of the conference have a lasting impact on the practice and research of plastic surgery, an examination of the presented content provides insight into the driving developments in plastic surgery in Germany.

Materials and methods: We conducted a retrospective network analysis of all abstract titles from the DGPRÄC and VDÄPC annual meeting in 2023. Data were extracted regarding titles, language, author, and place of origin, and the titles were categorized into the four pillars of the specialty. The titles were standardized and subjected to network analysis.

Results: A total of 299 titles from 281 lectures and 18 instructional courses were analyzed. After preprocessing the data, 2463 words with 9384 connections qualified for network analysis. The most frequently mentioned keywords throughout the congress were 'Surgery', 'Breast', 'Reconstruction', 'Flap', 'Patient', 'Tissue', and 'Therapy'. Locations contributing the highest number of abstracts were Ludwigshafen, Hanover, Leipzig, and Munich.

Conclusion: In the era of big data, network analysis provides the ability to identify underlying structures and nodes in multidimensional, complex datasets. This study demonstrates the useful application of network analysis to identify thematic focuses and connections at the current DGPRÄC and VDÄPC annual meeting. Sites of intensified research could thus be identified.

## Introduction

Plastic surgery is a diverse surgical specialty that includes various sub-disciplines. It has significant overlaps with other medical fields such as trauma surgery, oral and maxillofacial surgery, and general surgery. This interlinking nature allows for a comprehensive and multidimensional approach to patient care. Recent advancements in scientific research and surgical techniques have led to remarkable progress in the field [1,2]. As a result, patients with severe injuries or congenital deformities now have the opportunity to lead sufficiently normal and fulfilling lives [3,4].

The joint annual conference of the German Society for Plastic, Reconstructive and Aesthetic Surgery (DGPRÄC) and the Association of German Aesthetic Plastic Surgeons (VDÄPC) represents the largest German congress for the specialist field of plastic surgery. Clinical doctors, scientists, and industry professionals convene here to exchange the latest scientific findings through lectures, poster presentations, workshops, and courses. The congress covers all four pillars of plastic surgery: reconstructive surgery, hand surgery, aesthetic surgery, and burn surgery. Under the title 'Evidence, Eminence, Excellence', the current conference took place from September 14, 2023, to September 16, 2023, in the 'New University' of the time-honored Heidelberg 'Ruperto Carola' and attracted well over 1000 visitors from Germany, Europe, and the United States. A total of over 300 lectures, courses, and webinars were held [5].

Given this wealth of topics, it can be challenging to get an overview of the main content and themes of the current conference. This is where network analysis offers the possibility to evaluate complex, multidimensional data. As a result, network analysis has emerged in various fields, including the social and political sciences [6,7]. In recent years, medical research has begun to utilize this form of data analysis [8,9]. For instance, network analysis has aided psychopathology in gaining a better understanding of mental illnesses by identifying symptom dynamics and revealing comorbidities [10]. As recent studies in dermatology have shown [11,12], there are many more opportunities that need to be exploited, especially for the analysis of text-based datasets.

Due to the lasting influence of the joint annual meeting of the DGPRÄC and VDÄPC on the plastic surgery community, the aim of this study was to identify the leading topics of the conference by means of network analysis. The insights gained help to understand what is currently driving plastic surgery.

## Materials and methods

Data acquisition

We performed a retrospective analysis of all abstract titles presented at the joint annual meeting of the DGPRÄC and the VDÄPC in 2023. The information on the abstract titles, authors, and locations was obtained from the official program booklet [5]. The data were classified by title, language of presentation, author, city, country, and presentation format. In addition, the lectures and courses were categorized into one of the four pillars of plastic surgery. Topics that could not be categorized were placed in a fifth category called 'miscellaneous', which was further divided into six subcategories. All data were transferred to Microsoft Excel.

Preprocessing

Titles were preprocessed using Python (The Python Software Foundation, Wilmington, DE, USA) code in Jupyter Notebook (version 3.11.5). We used Python's Pandas (version 2.1.1), an open-source data analysis application, and Python's Natural Language Toolkit (version 3.2.5), a Python text processing library. All titles were translated into English to perform a semantically unambiguous network analysis of all keywords in the abstract titles. We used Googletrans (version 4.0.0rc1), a Google API for language translation. The titles were stripped of filler words, such as articles and pronouns, which were not keywords for the title analysis. To accurately capture multiple mentions of the same words, conjugation features were removed and singular and plural forms were aligned.

Network analysis

The network analysis was performed using Gephi (version 0.10.1), an open-source tool for visualizing and analyzing networks. A co-occurrence matrix of all keywords appearing together in a title was generated and the resulting network was analyzed. 

In network science, networks are composed of nodes and edges. The nodes were represented by the individual words of the abstract titles, while the edges corresponded to connections resulting from the co-occurrence of two words within a title. Node labeling and size were proportional to keyword frequency. Edge strength, indicated by the thickness of the connecting line between two nodes, was set in relation to the weight of the edge, i.e., the frequency of co-occurrence of two connected words. To ensure the clarity of the network composition for the entire conference, we assumed a minimum keyword frequency of n = 3. In the network composition for the subspecialty of reconstruction, we determined a minimum keyword frequency of n = 2. For all the other pillars, we did not use a frequency filter due to the lower number of keywords.

The network compositions were created using the Fruchterman Reingold layout [13]. For better clarity, the network parameters were manually adjusted, including minimum node size (10.0), maximum node size (100.0), area (10000.0), attraction force (20.0), node opacity (70.0), node label (80.0), edge thickness (3.0), and edge opacity (15.0). The edges were colored to match the nodes they connected, based on the determined modularity class. Nodes belonging to the same class were assigned the same randomly chosen color. Modularity, which measures the network's ability to be divided into distinct communities, was used as the basis for this determination [14]. It can be represented by the so-called Q-value, which ranges between -1 and 1. A highly modular network is characterized by strong connections within its compartments and weak connections between them [15].

The origin of the submissions was analyzed by extracting the coordinates of the cities using the OpenCage Geocoding library for Python [16]. The heat maps were created using the Folium library (version 0.15.0), a Python library for creating interactive maps. 

## Results

Title analysis

A total of 299 abstract titles were analyzed. These resulted from 281 lectures and 18 instructional courses, partly in German (n = 236; 79%) and partly in English (n = 63; 21%). The titles were assigned to the four pillars resulting in the following distribution: 110 titles (74%), consisting of 104 lectures and six instructional courses, focused on the topic of reconstructive surgery, which accounted for the majority of the 2023 annual meeting. Aesthetic surgery came in second with 66 titles (22%), comprising 62 lectures and four instructional courses. Twenty-seven abstracts (9.0%) were related to hand surgery, including 23 lectures and four courses, while 22 abstracts (7.4 %) focused on burn surgery (20 lectures and two courses). Seventy-four abstracts were categorized as 'miscellaneous' (24.7 %), comprising 27 lectures on policy (9.0 %), 19 lectures, and two courses on research (7.0 %), 10 lectures on gender reassignment surgery (3.3 %), nine lectures on continuing education (3.0 %), six lectures on social media (2.0 %), and one keynote lecture titled ‘Innovo ergo sum’ (0.3 %).

Network composition

**Figure 1:**
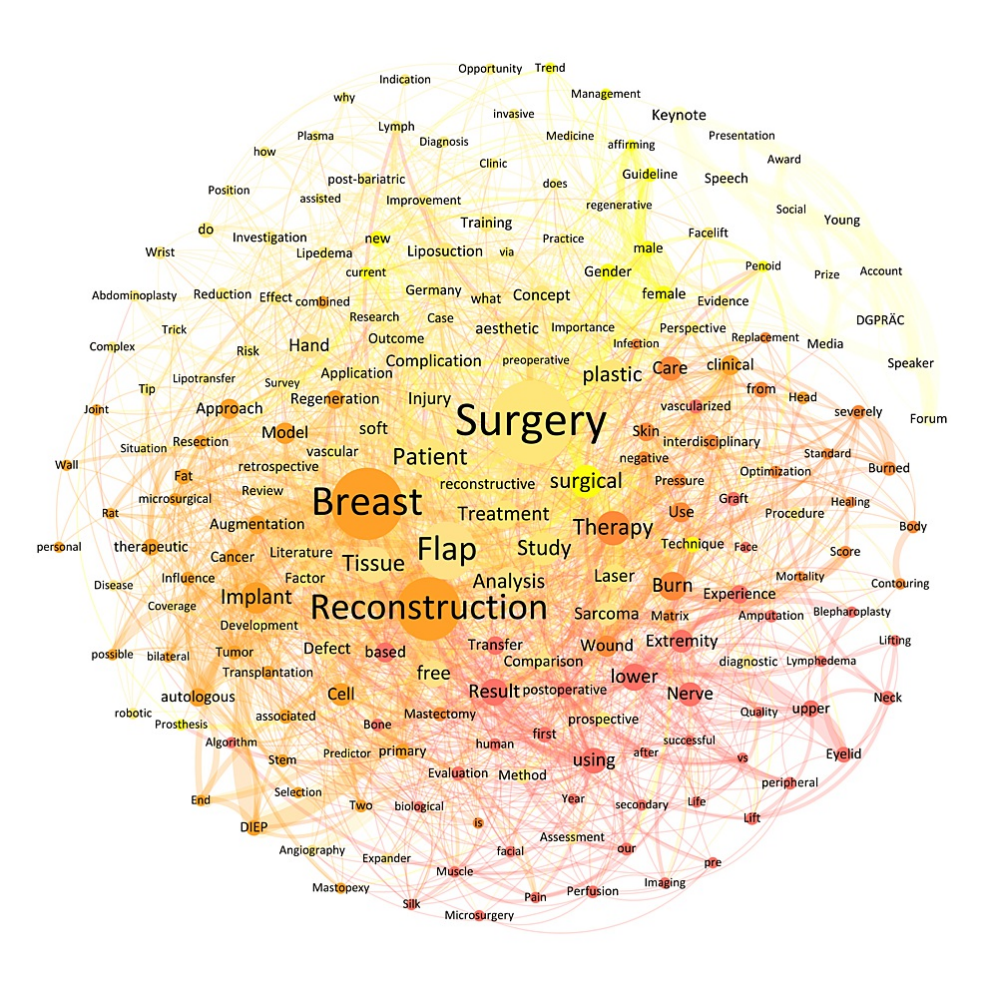
Weighted network composition (Fruchterman Reingold layout) of the most frequent keywords of the abstracts of the DGPRÄC and VDÄPC annual meeting 2023 with a minimum mention of n = 3. This corresponded to 208 nodes (18% visible) and 2263 edges (24% visible) as well as a modularity of 0.32 with seven communities found (nodes of the same color corresponding to the same community). DGPRÄC: German Society for Plastic, Reconstructive and Aesthetic Surgery; VDÄPC: Association of German Aesthetic Plastic Surgeons.

The 299 titles consisted of 2938 words. By preprocessing the titles, we extracted a total of 2463 words that qualified for network analysis. Multiple entries resulted in 1132 nodes. The keywords with the highest proportion relative to the total number of words across all topics were 'Surgery' (n = 53, 2.2%), 'Breast' (n = 45; 1.8%), 'Reconstruction' (n = 39; 1.6%), 'Flap' (n = 35; 1.4%), 'Patient' (n = 21; 0.8%), and 'Tissue' (n = 20; 0.8%) (Figure 1). Their frequent mention underscores the focus areas that are pivotal to both current practice and future advances in plastic surgery.

**Figure 2:**
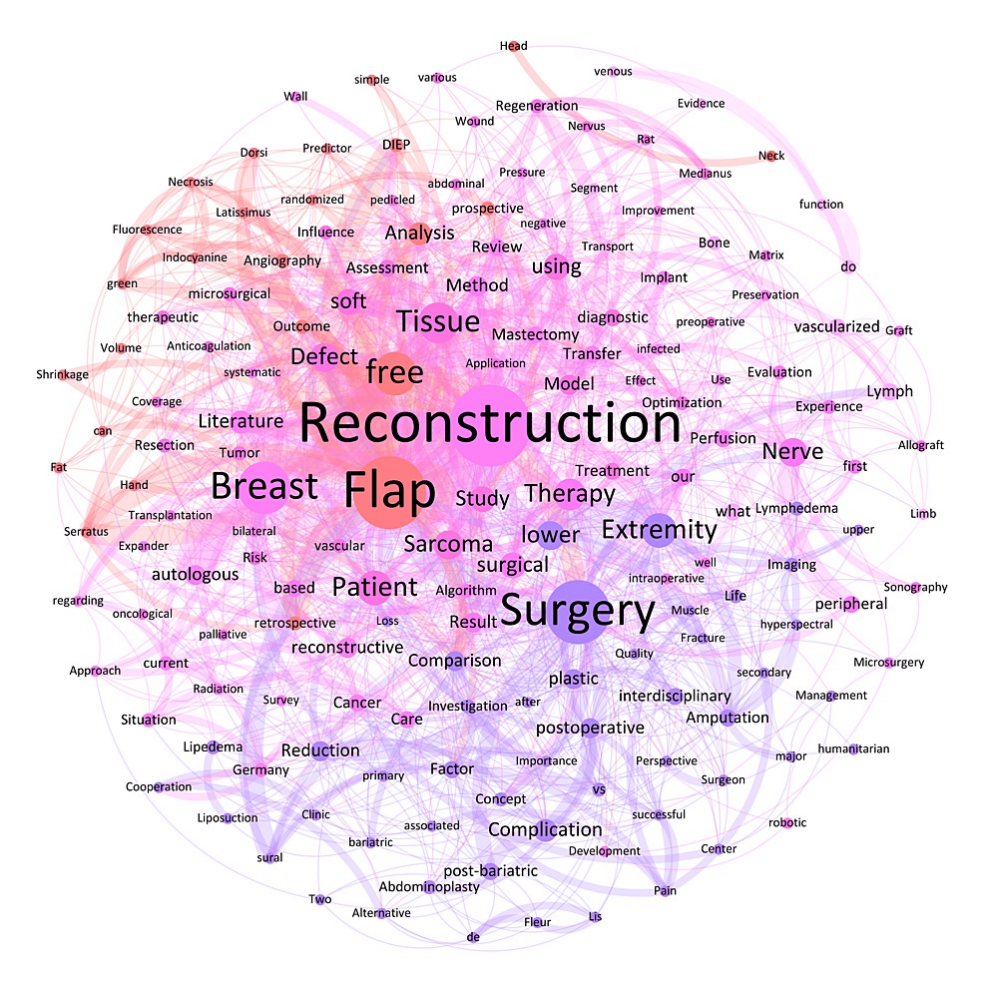
Weighted network composition (Fruchterman Reingold layout) of the abstract titles on reconstructive surgery with a minimum keyword mention of n = 2, corresponding to 170 nodes (34% visible) and 1418 edges (37% visible) as well as a modularity of 0.38 with seven communities found (same-colored nodes corresponding to keywords of the same community).

In the field of reconstructive surgery, the prominence of keywords such as 'Reconstruction' (n = 26; 2.8%), 'Flap' (n = 23; 2.4%), 'Surgery' (n = 20; 2.1%), 'Breast’ (n = 16; 1.7%), 'free' (n = 13; 1.4%), 'Tissue' (n = 12; 1.3%), and 'Extremity' (n = 10; 1.1%) highlights the ongoing focus and advancement in breast surgery and reconstruction (Figure 2). These findings align with recent literature that illustrates significant progress in breast reconstruction techniques, improving outcomes in terms of both aesthetics and functionality [17].

**Figure 3:**
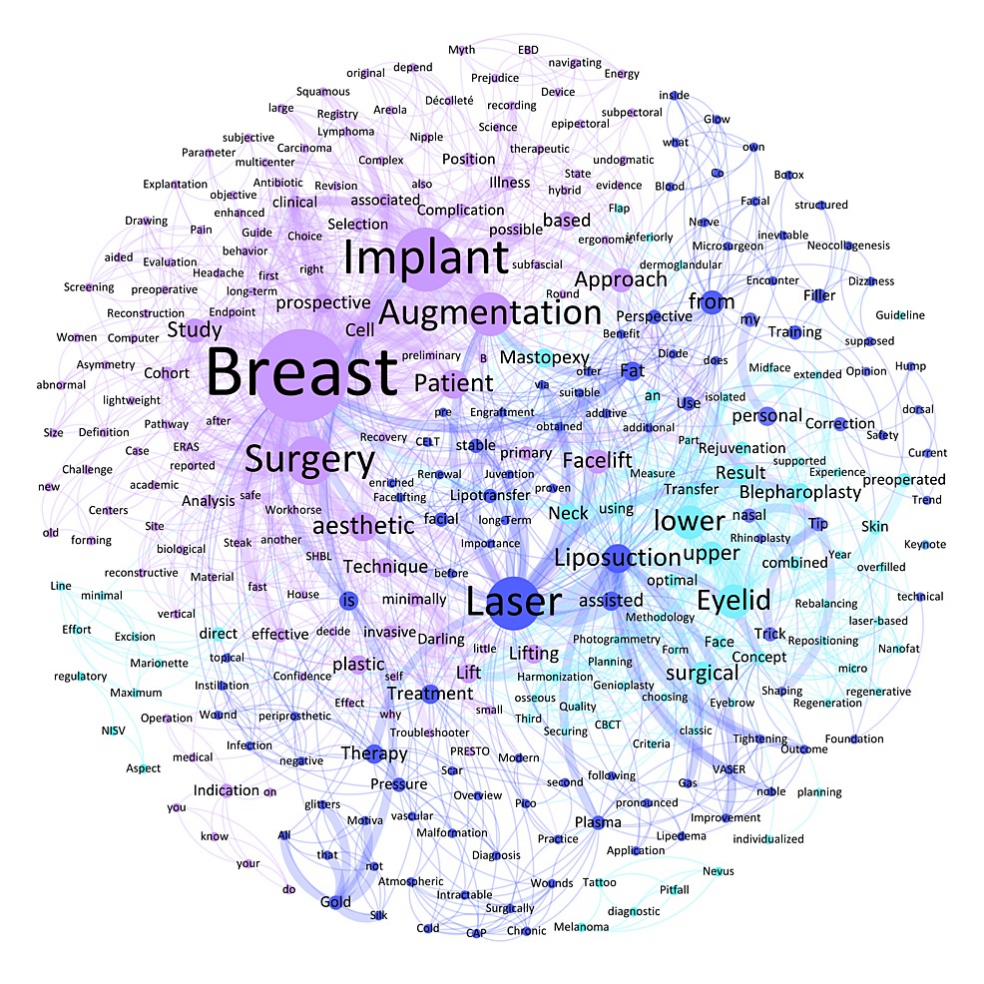
Weighted network composition (Fruchterman Reingold layout) of the abstract titles on aesthetic surgery, with 312 nodes and 1688 edges as well as a modularity of 0.52 with eight communities found (nodes of the same color corresponding to keywords of the same community).

In the aesthetic surgery category, the most frequently mentioned keywords relative to the total number of words analyzed (n = 479) were 'Breast' (n = 18; 3.8%), 'Implant' (n = 12; 2.5%), 'Laser' (n = 10; 2.1%), 'Surgery' (n = 9; 1.9%), 'Augmentation' (n = 8; 1.7%), 'Eyelid' (n = 6; 1.3%), 'lower' (n = 6; 1.3%), and 'Liposuction' (n = 5; 1.0%) (Figure 3), reflecting a surge in technological advancements and technique refinement. The mention of 'Laser' relates to innovations in laser therapy, such as fractional photothermolysis and photodynamic therapy, which are broadening treatment scopes and improving patient satisfaction with cosmetic outcomes. This underscores a trend towards more personalized, less invasive procedures that reduce recovery time and enhance aesthetic results.

**Figure 4:**
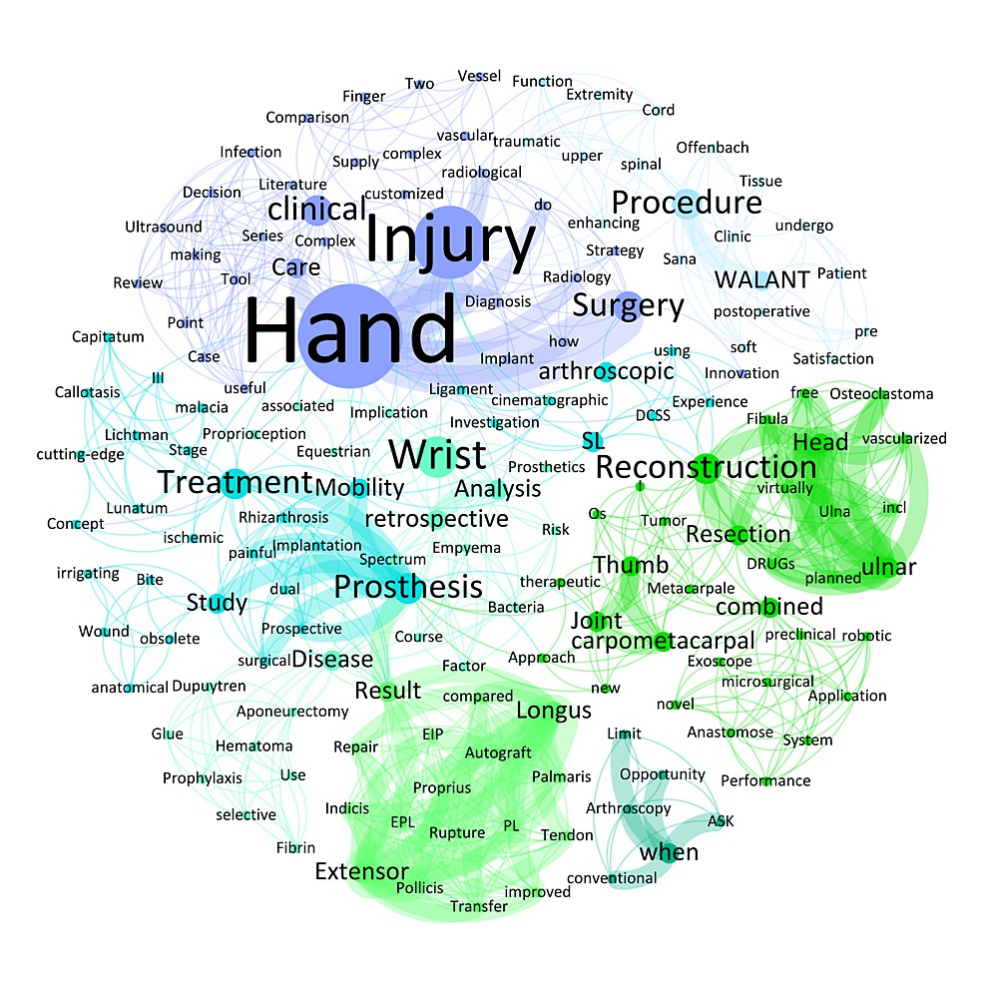
Weighted network composition (Fruchterman Reingold layout) of the abstract titles on hand surgery, with 164 nodes and 834 edges as well as a modularity of 0.76 with seven communities found (nodes of the same color corresponding to keywords of the same community).

Most frequently used keywords in the field of hand surgery, such as 'Hand' (n = 10; 4.7%), 'Injury' (n = 7; 3.3%), 'Wrist' (n = 4; 1.9%), 'Prosthesis' (n = 3; 1.4%), 'Mobility' (n = 3; 1.4%), 'arthroscopic' (n = 2; 0.9%), 'carpometacarpal' (n = 2; 0.9%), and 'WALANT' (n = 2; 0.9%) (Figure 4), indicate a focused interest in improving surgical outcomes through both new surgical techniques and innovations in anesthesia, reflecting a shift towards procedures that enhance intraoperative precision and postoperative recovery.

**Figure 5:**
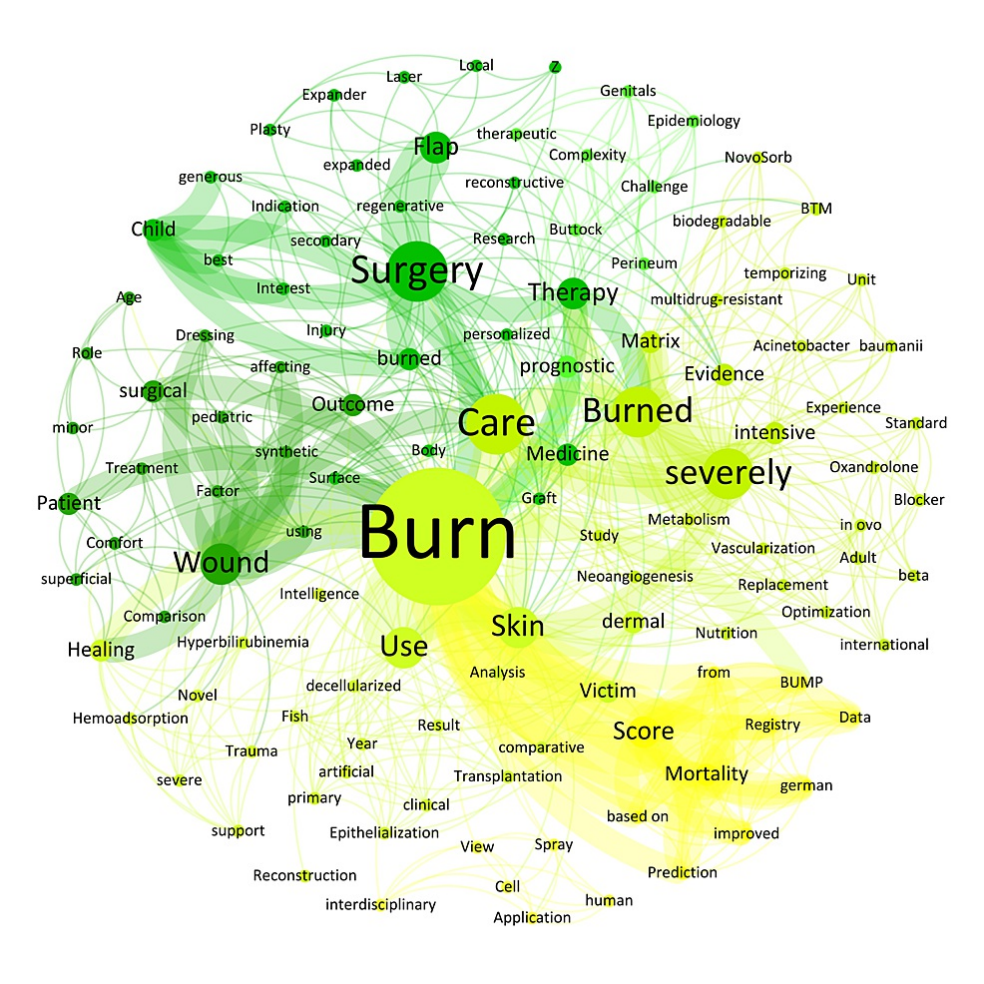
Weighted network composition (Fruchterman Reingold layout) of the abstract titles on burn surgery with 120 nodes and 653 edges. The modularity was 0.53 with seven communities found (nodes of the same color corresponding to keywords of the same community).

Similarly, in the category of burn surgery, the emphasis on keywords such as 'Burn' (n = 14; 7.8%), 'Surgery' (n = 6; 3.3%), 'care' (n = 6; 3.3%), 'severely' (n = 5; 2.7%), 'Wound' (n = 4; 2.2%), 'Skin' (n = 4; 2.2%), 'Score' (n = 3; 1.6%), and 'Matrix' (n = 2; 1.1%) demonstrates an ongoing commitment to optimizing therapeutic strategies, particularly through the use of advanced biomaterials and regenerative techniques which aim to improve healing processes and functional outcomes for severely burned patients​ (Figure 5).

Following pre-processing, 647 words were identified in the 'Miscellaneous' category, with keywords such as 'Gender' (n = 7; 1.1%), 'Keynote' (n = 7; 1.1%), 'Speaker' (n = 5; 0.8%), 'young' (n = 5; 0.8%), 'Forum' (n = 5; 0.8%), 'Media' (n = 5; 0.8%), and 'Guideline' (n = 4; 0.6%) being particularly prevalent.

The 1137 nodes of the total network showed 9384 edges. The most intensive connections across all conference titles were observed for the following keywords: 'Breast' and 'Reconstruction' (n = 24), 'free' and 'Flap' (n = 14), 'Flap' and 'Reconstruction' (n = 14), 'plastic' and 'Surgery' (n = 13), 'Breast' and 'Implant' (n = 12), and 'autologous' and 'Breast' (n = 12). By way of example, the keywords with the most connections within the four pillars were: 'Breast' and 'Implant' (n = 10) in the field of aesthetic surgery, 'severely' and 'Burned' (n = 5) in burn surgery, 'Hand' and 'Injury' (n = 5) in hand surgery and 'free' and 'Flap' (n = 14) in reconstructive surgery.

Location analysis

**Figure 6:**
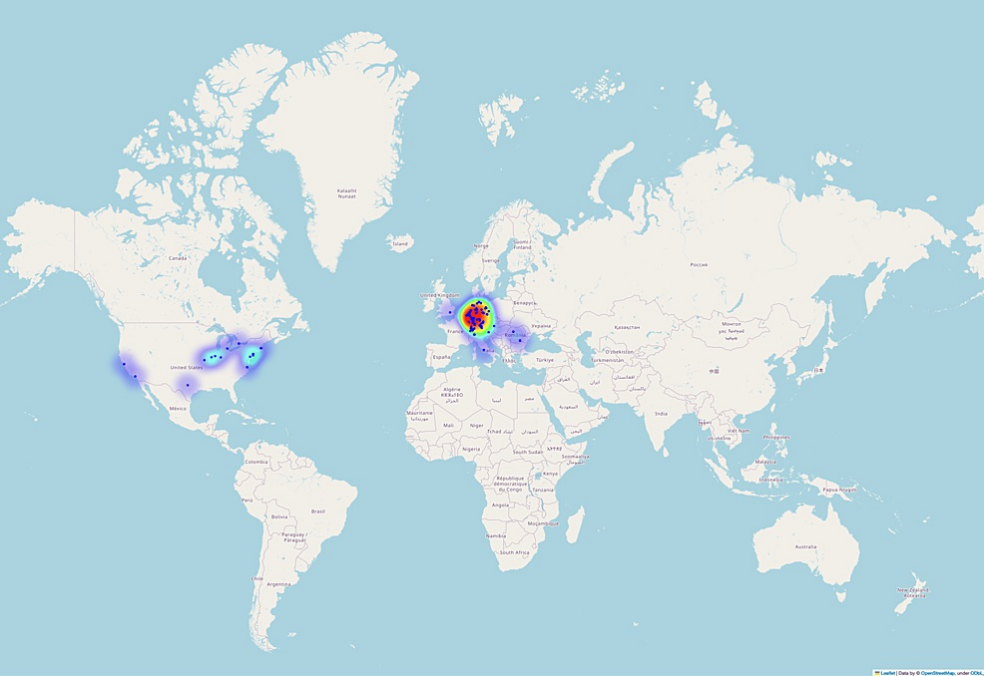
Worldwide heatmap of the original locations of the submitted abstracts of the DGPRÄC and VDÄPC annual meeting 2023. Sites were color-coded according to the number of submissions on a continuum from blue (few submissions) to red (many submissions). DGPRÄC: German Society for Plastic, Reconstructive and Aesthetic Surgery; VDÄPC: Association of German Aesthetic Plastic Surgeons.

Abstracts were submitted from 91 sites in nine countries (Figure 6). As expected for a German-language congress, the majority of submissions came from Germany (n = 241; 80.6%).

**Figure 7:**
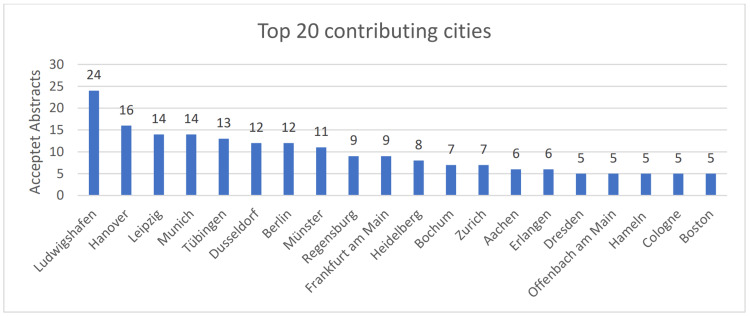
Bar chart of the locations with the most contributions to the DGPRÄC and VDÄPC annual meeting 2023 by number of accepted abstracts. DGPRÄC: German Society for Plastic, Reconstructive and Aesthetic Surgery; VDÄPC: Association of German Aesthetic Plastic Surgeons.

Additionally, 18 of the top 20 submitting locations were in Germany (Figure 7), highlighting the central role of German institutions in shaping the conference discourse. On the other hand, the location analysis revealed significant international contributions, indicating the global relevance of the research topics discussed. The USA submitted 25 titles (8.4%) and Switzerland submitted 21 titles (7.0%). Three titles came from Romania (n = 3; 1.0%), two from Italy (n = 2; 0.7%), one each from Canada (n = 1; 0.3%), Austria (n = 1; 0.3%), Spain (n = 1; 0.3%), and the United Kingdom (n = 1, 0.3%).

**Figure 8:**
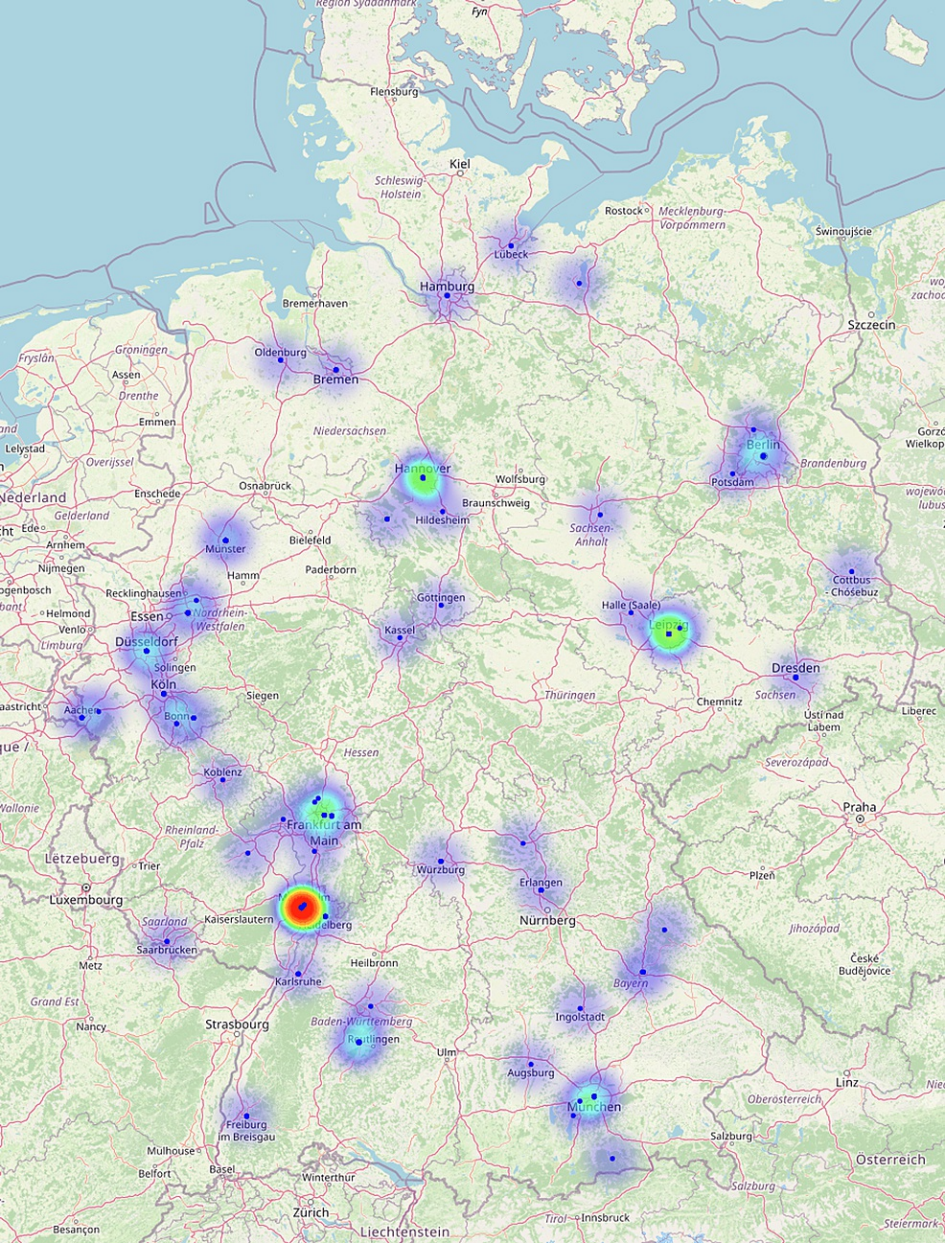
Heatmap of the locations of the submitted abstracts for the DGPRÄC and VDÄPC annual meeting 2023 in Germany. Sites were color-coded according to the number of submissions on a continuum from blue (few submissions) to red (many submissions). DGPRÄC: German Society for Plastic, Reconstructive and Aesthetic Surgery; VDÄPC: Association of German Aesthetic Plastic Surgeons.

Within Germany, the sites with the most abstracts were Ludwigshafen (n = 24), Hanover (n = 16), Leipzig (n = 14), Munich (n = 14), and Tübingen (n = 13) (Figure 7). In total, there were contributions from 57 different German cities (Figure 8). Multiple submissions from larger sites were partly due to the fact that several departments within a site contributed to the conference.

## Discussion

The network analysis of the DGPRÄC and VDÄPC annual meeting in 2023 revealed several topic clusters related to the four pillars of plastic surgery and provided an insight into research-intensive locations in Germany and abroad. In terms of the relative proportion of contributions, reconstructive surgery was the frontrunner at the current annual conference. The main topics discussed were free flap surgery and breast reconstruction. In recent years, developments in the field of free flap surgery have led to considerable improvements in postoperative monitoring, microsurgical techniques, patient-specific planning, and the associated reconstruction options. Although free flap surgery remains one of the most challenging tasks for the plastic surgeon, these advances are contributing to a steady expansion of reconstruction options. In the last ten years, various monitoring technologies have been developed for image-guided, postoperative monitoring of free flaps in order to establish an objective and reliable method of monitoring in the future [17]. Imaging techniques continue to be increasingly used for the preoperative planning of free flap transfers, particularly for the virtual mapping of perforators [18]. This improves patient-specific planning and microsurgical outcomes. Finally, an improved understanding of microvascular anatomy has led to the advent of free-style flap plasty, a form of reconstruction that has significantly expanded the range of potential donor sites and led to a paradigm shift in reconstructive surgery by departing from traditional flap plasty [19,20].

Remarkable progress has also been made in the development of breast reconstruction. Improvements in autologous and implant-based reconstruction now allow for refined aesthetic results and optimized restoration of breast sensation [21]. This includes improvements in the treatment of lymphedema, the development of acellular dermal matrices, improved breast implants, the introduction of prepectoral reconstruction, and the trend toward the preservation of the nipple-areola complex [22]. As a result, treatment options have expanded and patient satisfaction has increased despite the continued rise in breast cancer incidence rates [23].

With a relative share of 22.1 % of contributions, aesthetic surgery was the second largest pillar at the annual conference. Laser therapy, a frequent topic of discussion at the meeting, is experiencing a steady upswing due to expanded applications and new resurfacing techniques. The expanded areas of application today include fractional photothermolysis for skin rejuvenation, photodynamic therapy for acne treatment, and radiofrequency therapy to reduce the laxity in stressed skin areas [24]. Novel laser resurfacing techniques offer significant advantages over conventional ablative procedures such as CO2 and Erbium YAG (yttrium-aluminum-garnet) laser procedures in terms of complication rates and shorter recovery times [25]. In addition, new devices combine ablative and fractional procedures and can achieve remarkable results [26], for example in the treatment of facial scars [27].

Hand surgery and burn surgery were approximately equal in terms of contributions. Among other topics, the WALANT (Wide Awake Local Anesthesia No Tourniquet) procedure was mentioned several times in the field of hand surgery [28]. Since the concept was introduced by Dr. Donald H. Lalonde in the early 2000s, new applications for the technique in hand surgery have been steadily developed [28]. In addition to well-known indications such as carpal roof splitting or tendon surgery, recent studies have also demonstrated its safe use for more extensive soft tissue reconstructions and fracture treatment [29]. Due to the impressive advantages of eliminating the tourniquet, increased patient safety by dispensing with general anesthesia, and the possibility of patient cooperation during the procedure, the procedure is expected to become more established in the years to come.

Finally, the contributions from the field of burn surgery addressed issues related to specific intensive medical care and the still challenging question of optimal coverage of severely burned skin areas. The application and testing of various dermal skin substitutes (e.g., Suprathel®), different wound dressings and matrices (e.g., epicite® hydro, Novosorb® BTM), personalized skin grafts or decellularized fish skin continues to be a broad and innovative field of research, as demonstrated by the diversity of burn presentations at the recent DGPRÄC and VDÄPC annual meeting.

The visualization of the contributing locations was able to outline regional differences and highlight some heavyweights in plastic surgery research. Although the majority of contributions came from Germany, the significant participation from other countries, including the USA and Switzerland, demonstrates the international reach of the conference.

Limitations of the study arise, on the one hand, from the assumption that the authors have described the content of the articles sufficiently in the title of the abstracts. This may not always have been the case. If an author deliberately does not state the name of a procedure or disease that is the focus of an article, but instead uses a vague title, a qualitative classification using network analysis is not possible. Data standardization also works differently for different subject areas. It is particularly difficult when there are many different names or reference topics for a procedure or pathology. Second, the generalizability of the results of this study is limited. Not all relevant scientists and surgeons contribute to the DGPRÄC and VDÄPC annual meeting, even if they are driving decisive developments in the field. Therefore, the topics of the annual conference only incompletely reflect the true state of research in plastic surgery.

## Conclusions

This study utilized network analysis to distill the complex and multifaceted data presented at the German Society of Plastic, Reconstructive and Aesthetic Surgery (DGPRÄC) and Association of German Aesthetic Plastic Surgeons (VDÄPC) 2023, revealing prevalent research and practice trends in German plastic surgery. We found that 'Breast,' 'Reconstruction,' and 'Flap' were among the most frequently mentioned terms, highlighting the ongoing emphasis on advanced reconstructive techniques and their critical role in patient care outcomes. The analytical approach enabled the identification of central themes and illustrated the network of interactions between various topics, reflecting the dynamic nature of the field. Furthermore, our analysis pinpointed locations with concentrated research activity, providing insights into geographic and institutional contributions to plastic surgery in Germany. These findings not only showcase the current state of plastic surgery but also serve to guide future research directions and collaborations.

By mapping the intellectual landscape of the DGPRÄC and VDÄPC annual meeting, this paper underscores the potential of network analysis as a powerful tool to elucidate emerging trends and focal points in medical specialties. The implications of these findings are significant, offering a roadmap for future conferences and research initiatives to enhance the development of the field and ultimately improve patient care. This study exemplifies the integration of digital analysis tools in medical research, promising further innovations in understanding complex datasets and fostering a data-driven approach to medical science.
